# Regulation of calretinin in malignant mesothelioma is mediated by septin 7 binding to the *CALB2* promoter

**DOI:** 10.1186/s12885-018-4385-7

**Published:** 2018-04-27

**Authors:** Walter Blum, László Pecze, Janine Wörthmüller Rodriguez, Martine Steinauer, Beat Schwaller

**Affiliations:** Anatomy, Department of Medicine, University of Fribourg, Route Albert-Gockel 1, CH-1700 Fribourg, Switzerland

**Keywords:** Calretinin, Septin 7, Malignant mesothelioma, Transcriptional regulation, Butyrate

## Abstract

**Background:**

The calcium-binding protein calretinin (gene name: *CALB2*) is currently considered as the most sensitive and specific marker for the diagnosis of malignant mesothelioma (MM). MM is a very aggressive tumor strongly linked to asbestos exposure and with no existing cure so far. The mechanisms of calretinin regulation, as well as its distinct function in MM are still poorly understood.

**Methods:**

We searched for transcription factors binding to the *CALB2* promoter and modulating calretinin expression. For this, DNA-binding assays followed by peptide shotgun-mass spectroscopy analyses were used. *CALB2* promoter activity was assessed by dual-luciferase reporter assays. Furthermore, we analyzed the effects of *CALB2* promoter-binding proteins by lentiviral-mediated overexpression or down-regulation of identified proteins in MM cells. The modulation of expression of such proteins by butyrate was determined by subsequent Western blot analysis. Immunohistochemical analysis of embryonic mouse lung tissue served to verify the simultaneous co-expression of calretinin and proteins interacting with the *CALB2* promoter during early development. Finally, direct interactions of calretinin with target proteins were evidenced by co-immunoprecipitation experiments.

**Results:**

Septin 7 was identified as a butyrate-dependent transcription factor binding to a *CALB2* promoter region containing butyrate-responsive elements (BRE) resulting in decreased calretinin expression. Accordingly, septin 7 overexpression decreased calretinin expression levels in MM cells. The regulation was found to operate bi-directionally, i.e. calretinin overexpression also decreased septin 7 levels. During murine embryonic development calretinin and septin 7 were found to be co-expressed in embryonic mesenchyme and undifferentiated mesothelial cells. In MM cells, calretinin and septin 7 colocalized during cytokinesis in distinct regions of the cleavage furrow and in the midbody region of mitotic cells. Co-immunoprecipitation experiments revealed this co-localization to be the result of a direct interaction between calretinin and septin 7.

**Conclusions:**

Our results demonstrate septin 7 not only serving as a “cytoskeletal” protein, but also as a transcription factor repressing calretinin expression. The negative regulation of calretinin by septin 7 and vice versa sheds new light on mechanisms possibly implicated in MM formation and identifies these proteins as transcriptional regulators and putative targets for MM therapy.

**Electronic supplementary material:**

The online version of this article (10.1186/s12885-018-4385-7) contains supplementary material, which is available to authorized users.

## Background

The Ca^2+^-binding protein calretinin (CR) serves as an undisputed marker for the diagnosis of human malignant mesothelioma (MM), in particular of the epithelioid type and the epithelioid parts of the mixed type [1, 2]; weak CR expression is also found in sarcomatoid MM, possibly indicating a less important and/or different role of CR in this MM cell type [3]. CR is also expressed in human reactive mesothelial cells [4, 5], considered as the first step in the transition from healthy flat mesothelial cells covering the pleural cavities to one of the most aggressive and currently therapy-resistant tumor type. Down-regulation of CR by *CALB2* shRNA in human MM cell lines profoundly decreases cell growth and viability in vitro: lentivirus-mediated delivery of shCALB2 causes MM cells, in particular the ones with an epithelioid morphology, to enter apoptosis within 72 h post-infection [3]. Under these conditions, the intrinsic caspase 9-dependent pathway is activated. Although the immortalized mesothelial cells LP9/TERT1 show strong CR expression (3), shRNA-mediated CR down-regulation differently affects these non-transformed cells: it inhibits cell proliferation as the result of a G_1_ block. Neither is the viability impaired nor any type of cell death pathway activated.

CR is a fast Ca^2+^ buffer protein [6, 7] modifying the shape of intracellular Ca^2+^ transients [8]; overexpression of CR reduces the mitochondrial Ca^2+^ uptake in primary mesothelial cells [9]. Very little is known about the regulation of CR expression in the various tissues, even in the subpopulation of neurons, where CR is expressed under physiological conditions. It is assumed that CR expression is regulated in a rather similar way in humans and in mice, mostly based on the strong conservation of the proximal promoter regions of the human *CALB2* and mouse *Calb2* genes [10]. An AP2-like element in proximity of the TATA box confers neuron-specific expression of a luciferase reporter gene (*luc+*) in cultured neurons [11] (for additional details, see Fig. 1 in [12]), necessitating the binding of a nuclear protein present in cerebellar granule cells. Of note, this “AP2-like” element in the promoter region has no effect on the transcriptional activity in either MM cells or CR-expressing human colon cancer cells. Down-regulation of β-catenin by its negative regulator Axin2 significantly reduces CR expression in cultured rat thalamic neurons indicating that β-catenin is a positive regulator of the *Calb2* gene [13]. A more detailed *CALB2* promoter analysis revealed the sequence embracing the − 161/+ 80 bp region to sustain transcriptional activity in MM cells. Cis-regulatory elements within this promoter region including binding sites for NRF-1 and E2F2 are important for CR expression; e.g. siRNA-mediated down-regulation of NRF-1 causes a decrease in CR expression levels indicating that NRF-1 acts as a positive regulator of CR expression (14). Moreover, the strong correlation between *CALB2* mRNA and CR protein expression levels in MM cells is indicative of a control at the transcriptional level [14]. In colon cancer cells, two butyrate-responsive elements (BRE) embracing the TATA box of the *CALB2* gene function as butyrate-sensitive repressors of CR expression, while the same sequence has no effect in cells of mesothelial origin, e.g. Met-5A cells [15]. Butyrate (Bt) is the product resulting from intestinal fermentation of dietary fibers by bacteria and Bt concentrations in the range of 5–30 mM are present in the chyme/feces of the gut [16]. Bt acts as a modulator of histone acetylation that results in the inhibition of the cell cycle (G_1_ arrest) and leads to enterocyte (and derived cancer cells) differentiation [17]. Bt exposure of CR-expressing WiDr colon cancer cells results in CR down-regulation [18]. Moreover, gut microbiota might have an influence on respiratory infections [19] also via short chain fatty acids (SFCA) including Bt. Bt is not only produced by the gut microbiota, but also by anaerobic bacteria in the hypoxic environment of cystic fibrosis (CF) airways. Bt concentrations were found to be elevated in the sputum samples of CF patients reaching values of approximately 2 mM [20] and it is conceivable that in the diseased lung of MM patients, Bt may also be increased in the pleural cavity.

**Fig. 1:**
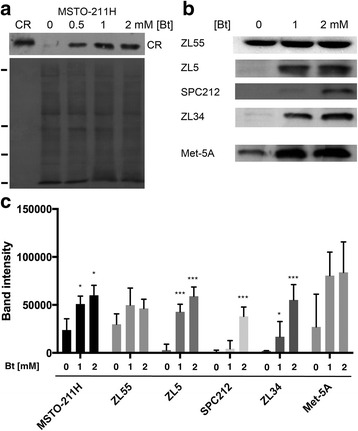
Upregulation of CR in Bt-exposed immortalized mesothelial (Met-5A) and MM cells **a** Upper panel: CR Western blot of cytosolic proteins from MSTO-211H cells treated with Bt for 72 h; left lane (CR): human recombinant CR (40 ng) as control. Lower panel: Ponceau Red-stained membrane to check for even loading. Ticks on the left mark the position of marker proteins (in kDa, from top to bottom: 75, 35, 28, 10). The signal for CR is slightly above the 28-kDa marker protein. **b** Western blots for CR of Bt-treated (72 h) cells: one mesothelial cell line (Met-5A) and 4 MM cell lines: ZL55, ZL5, SPC212, ZL34. Equal loading was confirmed by Ponceau Red staining as shown in **a** (not shown). **c** Semi-quantitative CR Western blot results (Mean ± S.D.) from 5 independent representative experiments. *** *p* < 0.001

In the present study, short-term exposure of MM-derived cell lines to low millimolar Bt concentrations revealed a significant increase in CR expression levels. Thus, we set out to investigate in more detail the promoter region of the *CALB2* gene that acts as a Bt-responsive enhancer in MM cells. To address the question on mechanisms implicated in this regulation, we searched for proteins binding to this particular promoter region that might be functionally implicated in CR regulation in MM cells.

## Methods

### Cell culture

The human mesothelioma cell lines MSTO-211H, H28, H226, the immortalized mesothelial cell line Met-5A and the colorectal adenocarcinoma HT-29 were obtained from the American Type Cell Collection (ATCC, Rockville, MD). The human mesothelioma cell lines ZL5, ZL34, ZL55, SPC111 and SPC212 were obtained from the University Hospital of Zurich (Switzerland) described by Schmitter et al. [21]. Cells were maintained in RPMI1640 (Gibco, Basel, Switzerland) supplemented with 10% fetal bovine serum (FBS, Gibco) and 100 U/ml penicillin and 100 μg/ml of streptomycin (1% PS, Gibco). LP9/TERT-1 cells were obtained from the laboratory of Dr. James Rheinwald (Dana Farber Cancer Research Institute, Boston, MA) and were maintained in a medium consisting of 1:1 M199 and MCDB10 medium supplemented with 15% newborn calf serum, 5 ng/mL epidermal growth factor, 0.4 μg/mL hydrocortisone, 2 mM glutamine, 100 U/ml penicillin and 100 μg/ml streptomycin (1% PS) (Gibco, Switzerland).

### Generation of reporter plasmids including deletion variants of the human *CALB2* promoter containing BRE7–13

**Fig. 2:**
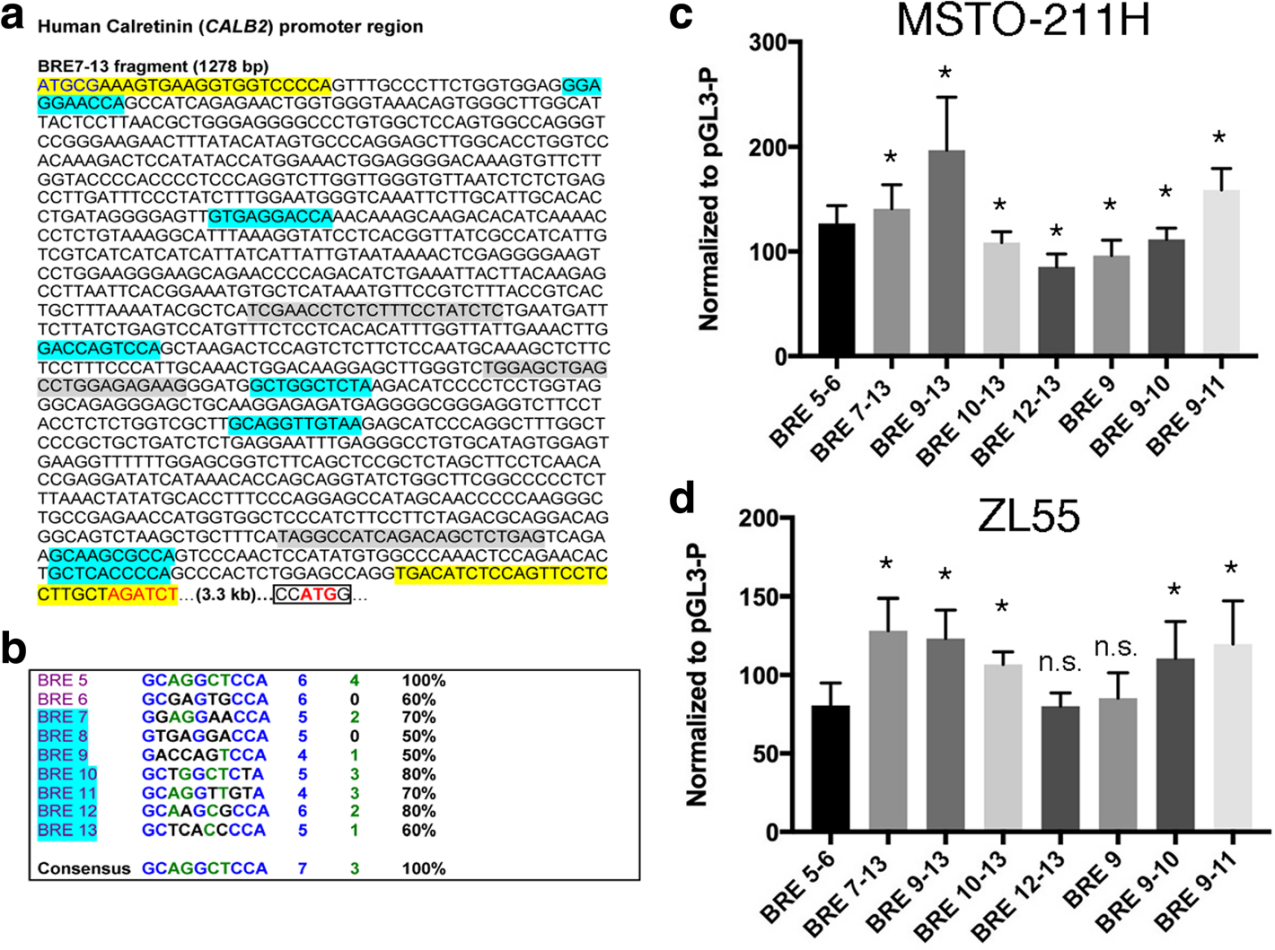
Sequence of the human *CALB2* promoter region containing putative Bt-responsive elements BRE7–13 **a** The region of 1278 bp contains 7 putative BREs (cyan). Primer sequences to amplify the 1278-bp fragment are marked in yellow including restriction sites for *SacI* (Table 1) and *BglII* (red). The start site of translation is marked in red (bold). Primers used to amplify truncated versions are boxed in gray (details are shown in Table 1). **b** Sequence alignment of BRE5 - BRE13 with the BRE consensus sequence (15) shown at the bottom. Nucleotide sequences fully complying with the consensus (100% identity) are marked in blue, medium-conserved sequences are marked in green and nucleotides not conferring to the consensus sequence are marked in black; percentages of identity with the consensus sequence are given in the right lane. **c**, **d** *Luc +* reporter assay in MSTO-211H (upper) and ZL55 MM (lower) cells with *CALB2* promoter fragments containing variable numbers of BREs. *Luc +* activities were normalized to the signal obtained with the control plasmid pGl3-P. As reference for the statistical analysis, BRE5–6 [15] was used. The number of independent experiments ranged from *n* = 4 (e.g. BRE10–13) to *n* = 11 (e.g. BRE7–13). Values represent mean ± S.D. *p < 0.05 vs. BRE5–6

Genomic DNA was isolated from MSTO-211H and ZL55 MM cells using standard methods and purified DNA (0.5 μg) was used as template to amplify a 1.3 kb stretch of the human *CALB2* promoter embracing the putative Bt-responsive elements (BRE) 7–13; the sequence and details of primers are shown in Fig. 2 and Table 1. The amplicon of 1292 bp containing a SacI and a BglII site at the 5′- and 3′-end, respectively, was cloned into the plasmid pGL3-Promotor (pGL3-P; Promega; # U47298; Wallisellen, Switzerland) linearized with the same restriction enzymes. pGL3-P contains a multiple cloning site, followed by a minimal SV40 promoter; the luciferase cassette (luc) in the plasmid is followed by a SV40 late poly (A) signal. This plasmid was then used to generate shorter PCR fragments containing a various number of BREs (see Fig. 2 and Table 1), the fragments were cloned into pGEM-TEasy, sequenced (Microsynth AG, Balgach, Switzerland) and excised by SacI and BglII. The fragments were then inserted into pGL3-P for transfection experiments; DNA (300–500 μg) was isolated using the Wizard Plus Midi- or Maxiprep kit (Promega, Dübendorf, Switzerland).

**Table 1:** All 5′ primers contain a *SacI* and all 3′ primers a *BglII* site for cloning into pGl3-P vector

Name BRE	5′ primer (5′-3′)	3′ primer (5′-3′)	size (bp)
7–13	GATGAGCTCATGCGAAAGTGAAGGTGGTCCCCAG	TGAAGATCTAGCAAGGAGGAACTGGAGATGTCA	1278
9–13	GAGCTCTCGAACCTCTCTTTCCTATCTC	AGATCTAGCAAGGAGGAACTGGAGATGTCA	695
10–13	GAGCTCTGGAGCTGAGCCTGGAGAGAAG	AGATCTAGCAAGGAGGAACTGGAGATGTCA	530
12–13	GAGCTCTAGGCCATCAGACAGCTCTGAG	AGATCTAGCAAGGAGGAACTGGAGATGTCA	127
9	GAGCTCTCGAACCTCTCTTTCCTATCTC	AGATCTCTTCTCTCCAGGCTCAGCTCCA	189
9–10	GAGCTCTCGAACCTCTCTTTCCTATCTC	AGATCTCCCTCATCTCTCCTTGCAGCT	253
9–11	GAGCTCTCGAACCTCTCTTTCCTATCTC	AGATCTAGGCCCTCAAATTCCTCAGAG	348

### Luciferase assay

In order to investigate *CALB2* promoter activity the dual luciferase assay was used. The constructs were co-transfected with Renilla-Luciferase in 12-well plates (50,000 cells/well) with Mirus lipofection reagent (Mirus, WI, USA). After 48 h (± Bt treatment; 1 mM) the cells were lysed and promoter activity was measured with the dual-luciferase reporter assay (Promega, Dübendorf, Switzerland) on a Turner Designs TD-20/20 Luminometer (Sunnyvale, CA, USA) according to the manufacturer’s protocol. The details on the normalization of signals and the way to calculate fold changes compared to untreated controls have been described before [15].

### DNA binding assay

The fragment containing BRE9–13 (706 bp) serving as capture DNA was synthesized by PCR using 5′-biotinylated primers. The μMACS™ FactorFinder kit (Mylteni Biotech, Bergisch Gladbach, Germany) was used to isolate proteins interacting with this stretch of the *CALB2* promoter. The cleared cell lysate (15,000 x g, 5 min) from 10^7^ MSTO-211H cells (100 μl) was incubated with 1.5 μg of biotinylated BRE9–13 DNA. Putative transcription factor complexes were isolated under non-denaturing (native) conditions according to the manufacturer’s protocol.

### Western blot analysis and silver staining

Cell pellets were collected and washed 3 times with CMF-PBS. Cytosolic fractions were isolated as described before [22] and the concentration of proteins was determined using the Bradford method (Bio-Rad, Hercules, CA). Samples were loaded and separated by SDS-PAGE (10% PAA) and subsequently transferred onto nitrocellulose membranes (Bio-Rad) by a semidry system (Witec, Litau, Switzerland). Equal protein loading was controlled by transient Ponceau S staining (Sigma) of the membranes. The primary antibodies against calretinin (CR7699/4; Swant, Marly, Switzerland) and septin 7 (rabbit polyclonal anti-septin 7; Bethyl Laboratories Inc., Montgomery, TX, USA or Millipore Corp. #ABT354, Temecula, CA, USA) were used at a dilution of 1:5000 overnight at 4 °C; anti-GAPDH was from Sigma (ref. G9545) and used at a working dilution of 1:10,000. Rabbit secondary antibody directly linked to horseradish peroxidase (Sigma-Aldrich) was diluted 1:10,000 and membranes were incubated for 2 h at room temperature. The detection was performed with the chemiluminescent reagent Luminata Classico or Forte (EMD Millipore Corporation, Billerica, MA, USA) and data were collected on an imaging system from Cell Biosciences (Santa Clara, CA, USA).

For normalization and quantification of Western blots, densitometric analysis of the Ponceau S-stained membranes was performed using the GeneTools software (Syngene, Cambridge, UK). The integral of the signals of all transferred proteins was previously shown to represent a more reliable way of normalization, since this reduces the bias towards a specific protein (e.g. GAPDH, α-actin) often used for normalization. Levels of reference (housekeeping) proteins were shown to vary between tissues or different cell lines and might also be affected by the experimental manipulations [23, 24]. GeneTools software was also used for the quantification of the specific Western blots signals for CR, septin 7 and GAPDH. In the results section, most often only selected representative regions of the Ponceau S-stained membranes marked as loading control (L.C.) are shown, in particular when Western blot signals were analyzed qualitatively.

### Septin 7 cDNA cloning into pLVTHM backbone

A lentiviral system was used to overexpress septin 7. Briefly, the GFP cassette in pLVTHM (Addgene plasmid #12247) was replaced with the human septin 7 (*SEPT7*) cDNA using the SpeI and PmeI restriction sites. *SEPT7* cDNA was obtained from ZL55 cell total RNA. RNA was extracted using the Qiagen RNAeasy kit following the manufacturer’s instructions; cDNA was synthetized from 500 ng of total RNA using the QuantiTect Reverse Transcription kit (Qiagen). Septin 7 was amplified with the following primers: FW_hSEPT7 5′-AGT CGT TTA AAC ATG TCG GTC AGT GCG AGA TCC-3′ and RV_hSEPT7 5′-AGT CAC TAG TTT AAA AGA TCT TCC CTT TCT T-3′ containing the SpeI and PmeI restriction sites, respectively and the amplicon was inserted into the vector pLVTHM. The correct sequence of the plasmid was verified by colony PCR and subsequent sequencing of the insert and the novel plasmid was called pLV-hSEPT7.

### Lentiviral constructs

Lentivirus particles were produced as described before [3, 25]. Briefly, HEK293T cells were co-transfected by the CaPO_4_-method with 3 μg of the envelope plasmid pMD2.G-VSVG (Addgene plasmid #12259), 8 μg of the packaging plasmid psPAX2 (Addgene plasmid # 12260) and either 10 μg of the transfer plasmid pLV-CALB2 as described in [25] or the plasmid containing the shRNA for CALB2 as reported before [3]. Five validated clones of pLKO.1-shRNA SEPT7 plasmids were obtained from Sigma-Aldrich labeled 1–4 and 63 (according to the last digit of The RNAi Consortium (TRC) number). pLV-hSEPT7 was used to overexpress septin 7.

### Immunohistochemistry on sections of mouse embryos from E10.5

Mouse embryos were collected at embryonic day 10.5 (E10.5) from C57Bl/6J mice and fixed by immersion in 4% PFA for 72 h, dehydrated and embedded in paraffin. Sections (3 μm) were de-paraffinized and treated with Tris/EDTA (1 mM/0.1 mM, pH 9) for the antigen retrieval by heating the sections in a boiling water bath for 20 min. Hydrogen peroxide (0.3%; 20 min incubation) served to quench endogenous peroxidases and the tissue was permeabilized with 0.1% PBS Tween20 (5 min incubation) followed by blocking with PBS containing 2% BSA and 1% horse serum (20 min incubation). Sections were incubated with primary antibodies (anti-CR 7699/4, Swant, Switzerland, anti-septin 7, Bethyl Laboratories, USA) 1:500 overnight at 4 °C. Sections were incubated with secondary antibodies (1:200 dilution) at room temperature for 2 h. After DAB (Sigma-Aldrich) staining, sections were subjected to (weak) hematoxylin counterstaining. Slides were scanned using a whole-slide imaging system from Hamamatsu (Nanozoomer, 2.0-HT).

### Immunofluorescence

Cells were seeded on 12-mm glass coverslips and fixed for 15 min with 4% paraformaldehyde. Non-specific binding sites were blocked by incubation with TBS containing donkey serum (10%) for 1 h and coverslips were then incubated overnight at 4 °C with the following antibodies diluted in TBS 1X: goat polyclonal anti-CR (1:500; cat# CG1, Swant, Marly, Switzerland) and rabbit polyclonal anti-septin 7 (1:500; Bethyl Laboratories, USA). After washing, coverslips were incubated with secondary antibodies for 3 h at room temperature with the following secondary antibodies: Alexa Fluor 488-conjugated donkey anti-rabbit IgG (1:100, Jackson Immunoresearch Laboratories, West Grove, PA, USA) and Cy5-conjugated donkey anti-goat IgG (1:100; Jackson). Nuclear DNA was stained using DAPI (5 μg/ml; Molecular Probes, Eugene, OR) and coverslips were mounted with Hydromount solution (National Diagnostics, Atlanta, GA). Images were acquired using a Leica fluorescent microscope DM6000B (Wetzlar, Germany) equipped with a Hamamatsu camera C4742–95 (Bridgewater, NJ). For the cells treated with Bt, cells were seeded onto 12-mm glass coverslips pre-coated with Matrigel (Corning, NY, USA) and treated with 1 mM Bt for 48 h.

### Co-immunoprecipitation (co-IP)

MM cells at a confluence of 70–90% were washed with ice-cold PBS and lysed with 1 ml ice-cold Triton X-100 lysis buffer (150 mM NaCl, 1% Triton X-100, 50 mM Tris-HCl, pH 8.0) supplemented with protease and phosphatase inhibitors. Cell lysates were incubated on ice for 30 min and then centrifuged (10,000×*g*, 10 min at 4 °C). To pull down CR, 2–4 μg of a rabbit polyclonal anti-CR antiserum (CR7699/4; Swant) was added to the cleared lysate and incubated for 10 min at 4 °C. μMACS protein A MicroBeads suspension (100 μl; Miltenyi Biotec, Auburn, CA, USA) was added to the lysate and incubated at 4 °C for 30 min. Samples were loaded on MACS separation columns (Miltenyi Biotec) and subjected to magnetic immunoprecipitation. The columns were washed 3 times with a wash buffer (150 mM NaCl, 1% NP-40, 0.5% sodium deoxycholate, 0.1% SDS, 50 mM Tris-HCl, pH 8.0). Bound protein complexes were eluted in 50 μl of pre-warmed SDS gel loading buffer 1X (50 mM Tris-HCl, pH 6.8, 50 mM DTT, 1% SDS, 0.005% bromophenol blue, 10% glycerol) and subjected to Western Blotting for septin 7 as described above. A mouse monoclonal anti-Paxillin antibody (1:2000; BD Bioscience) was used as negative control.

### MTT assay

To assess the putative toxic effects of Bt in MM cells, the mitochondrial activity of living cells was determined by the 3-(4,5-dimethylthiazol-2-yl)- 2,5-diphenyltetrazolium bromide (MTT) quantitative colorimetric assay. MM cells (1500–3000 depending on the cell line) were seeded in 96-well plates. From a stock solution (1 M NaBt in PBS), different amounts were added to yield Bt concentrations from 0.33 to 5 mM. After 3 days of incubation with Bt, MM cells were subjected to the MTT assay by adding fresh medium containing 0.5 mg/ml MTT and incubating the plates for 90 min at 37 °C. The supernatant was discarded and DMSO (200 μl) was added to dissolve the formazan crystals. The absorbance at 570 nm was measured using a microplate reader (Infinite Pro200; Tecan Austria GmbH, Groedig, Austria). For each experiment the absorbance value of untreated (control) cells was defined as 1.0 (100%).

### Statistical analysis

The data are presented as mean ± standard deviation of multiple experiments (*n* ≥ 3). The statistics were performed using StatPlus (AnalystSoft) applying a one-way ANOVA followed by a *post*-*hoc* Tukey HSD test. Differences were considered as statistically significant if a *p*-value was < 0.05.

## Results

### Butyrate induces CR expression in MM cells and also in immortalized mesothelial cells

We analyzed the effect of Bt on CR expression levels in cells derived from all MM histotypes. Bt concentrations ranging from 0.5–2 mM applied for short periods (48 and 72 h) were tolerated to various degrees; higher Bt concentrations (generally ≥2 mM) and prolonged exposure (> 6 days) resulted in massive death of most of the investigated MM cell lines (data not shown). Immortalized mesothelial cells (Met-5A) and ZL34 cells derived from a sarcomatoid MM showed massive cell death already at 1–2 mM when treated for 6 days. In the few surviving Met-5A cells CR expression levels were insignificantly affected as reported before [15]. Here we investigated the effect of Bt on CR expression levels at 72 h, a time point only mildly affecting cell viability. A quantitative Bt concentration – cell proliferation/viability curve was determined for MSTO-211H, ZL55 and ZL5 cells, the lines used in most of the further experiments (Additional file 1: Figure S1). The weakest effect of Bt on the MTT signal intensity (cell proliferation/viability) was observed in ZL5 cells, intermediate in MSTO-211H cells and the strongest Bt-dependent effect was seen in ZL55 cells. The shape of the curves is not reminiscent of a typical sigmoidal toxicity curve, but rather for a growth-inhibiting effect of Bt, as had been observed before in Bt-treated colon cancer cells and immortalized LP9/TERT1 mesothelial cells (G_1_ arrest) [3, 26]. In support, only few floating (dead) cells were observed at the investigated concentrations and the selected time point (72 h). Bt treatment of MSTO-211H (biphasic) cells increased CR protein levels in a Bt concentration-dependent way (Fig. 1a). An increase in CR was also seen Met-5A cells, as well as in the epithelioid MM cell lines ZL5 and ZL55, the biphasic cell line SPC212 and the sarcomatoid cell line ZL34 (Fig. 1b). Generally, cells derived from epithelioid and biphasic MM are characterized by higher basal CR expression levels than cells derived from sarcomatoid MM, in particular ZL34 and SPC111 cells, the latter with very low basal CR expression levels [3, 14]. Bt treatment of all cell lines led to a concentration-dependent increase in CR levels; semi-quantitative evaluation of the CR signals of the cell lines shown in Fig. 1b are depicted in Fig. 1c. Of note, CR expression levels were determined in cells that remained attached to the cell culture dishes, i.e. viable cells and not the floating and/or dead cells in the culture medium. Since MM cell lines of the epithelioid and biphasic type were previously shown to be strongly affected by CR down-regulation, much more than MM cells derived from sarcomatoid tumors [3], further experiments were carried out with ZL5 and ZL55 (epithelioid) and MSTO-211H (biphasic) cells.

### A region in the *CALB2* gene promoter containing 7 putative BRE elements acts as a butyrate-responsive enhancer in MSTO-211H and ZL55 MM cells

Analysis of the human *CALB2* promoter region revealed additional putative BRE elements named BRE7–13 (Fig. 2b) upstream of the previously identified region that contains 2 functional BRE (BRE5–6), which act as transcriptional repressors in colon cancer cells [15]. The newly identified region has a length of 1278 bp and is approximately 3.3 kb upstream of the *CALB2* translation start site (ATG) (Fig. 2a). This 1.3 kb region was amplified by PCR using genomic DNA from ZL55 and Met-5A cells and primers containing specific restriction sites (forward primer: *SacI* site; reverse primer *BglII* site) compatible with the reporter luciferase plasmid pGl3-P. Sequencing revealed the inserts amplified from the 2 cell lines to contain alterations in the nucleotide sequence when compared to the sequence annotated in PubMed (*CALB2*; gene ID: 794). The three changes (1 point mutation (A/T), 2 deletions of either 1 or 2 nucleotides) were present in the sequences of all clones derived from both cell lines, ZL55 and Met-5A, excluding this being PCR artifacts (Additional file 1: Figure S2). The others were specific for each cell line, consisting of point mutations, 2 in the insert derived from ZL55 cells and 3 in the insert derived from Met-5A cells (Additional file 1: Figure S2). None of these changes directly involved the sequences of the putative BREs. Besides the *luc +* reporter plasmid containing all 7 putative BREs (BRE7–13), truncated versions were generated by PCR and these included plasmids containing BRE9–13, 10–13 12–13, 9, 9–10 and 9–11 (Fig. 2c). In MSTO-211H cells, luciferase activity was increased by 41 ± 16% (mean ± SEM) by the full-length fragment after Bt treatment for 48 h; however, strongest activation (200 ± 24% of control) was observed with the fragment containing BRE9–13, while elimination of BRE9 (fragment BRE10–13), resulted in a luciferase activity close to basal levels (109 ± 9%). In a shorter promoter fragment that included BRE9 (BRE9–11), activity was partially increased (159 ± 14%), but not to values as seen with the BRE9–13 plasmid. Thus, the essential enhancer/activator part appeared to be contained in the region embracing BRE9–11, but the region containing BRE12 and 13 further enhanced the luciferase activity, indicative that the various BREs contributed to the effects in a rather complex manner. Similar results were obtained in ZL55 cells, although the maximal effect in the presence of the BRE9–13 promoter fragment was of smaller magnitude (123 ± 18%). Thus, the effects were nearly identical qualitatively (Fig. 2c, d), yet the effect was approximately 4-times smaller in ZL55 cells. It is noteworthy that the effect on the luciferase activity was rather well correlated with the fold increase in CR expression levels after Bt treatment (Fig. 1). The increase in CR expression was + 290% in MSTO-211H cells and + 60% in ZL55 cells, i.e. an approximately 5-fold difference. It appears that in ZL55 cells, where CR levels are rather high in control conditions, the relative Bt-mediated increase in CR expression is smaller than in MSTO-211H cells characterized by a lower “basal” CR expression level [14]. We also tested the BRE7–13 and BRE9–13 promoter fragments containing the *luc +* reporter in the colon cancer cell line Caco-2 in order to see, whether the enhancer function also persisted in colonocytes. A small, yet insignificant increase of + 19 ± 16% was observed in the presence of the BRE9–13 fragment and similar results were also observed with HT-29 colon cancer cells (data not shown). This hints that the BRE9–13-containing promoter region in the *CALB2* gene acts as a Bt-activated enhancer, with a strong preference for MM cells.

### Identification of proteins binding to CALB2 promoter fragments BRE5–6 and BRE9–13 in a Bt-dependent manner

**Fig. 3:**
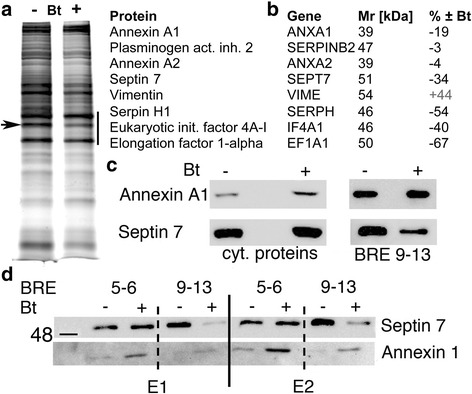
Proteins differentially bound to the BRE9–13 DNA fragment **a** Silver-stained gel of proteins eluted from the BRE9–13 DNA-containing column from control MSTO-211H cells (−) or from Bt-treated (1 mM) cells (+) for 72 h; the region between 35 and 50 kDa (black bar) showed the strongest signals. A band was clearly weaker in extracts from Bt-treated cells (arrow). **b** Differentially expressed proteins listed (from top to bottom) according to their abundance in the samples analyzed by peptide shotgun MS. **c** Western blot for annexin A1 (top) and septin 7 (bottom) of either total cytosolic proteins (left) or of the proteins bound to the BRE9–13 DNA (right). **d** Binding assay of MSTO-211H soluble whole cell extracts with either a DNA fragment containing BRE5–6 or BRE9–13 identified as the septin 7-binding region of the *CALB2* promoter. Eluate E1 was obtained after low stringency conditions, E2 under high stringency conditions

In a previous study we had demonstrated that both BRE5&6 contribute to the CR-repressing effect in colon cancer cells [15]. Based on the rather complex pattern of BRE-containing fragments on enhancing luciferase activity (Fig. 2c) involving up to 5 BREs [9–13], we addressed the question, which proteins might bind to the BRE9–13-embracing region in a Bt-dependent manner. For this, cell lysates from Bt-treated and control MSTO-211H cells were incubated with the biotinylated BRE9–13 DNA fragment. Several proteins bound to the DNA and were released in the presence of elution buffer (Fig. 3). A side-by-side comparison of silver-stained polyacrylamide gels revealed few proteins bands differentially bound to the BRE9–13 DNA fragment in the 2 conditions (± 1 mM Bt for 72 h). In the M_r_ range of 35–50 kDa several strongly stained bands were excised, digested by trypsin and analyzed by peptide shotgun mass spectroscopy (MS). Highly expressed proteins binding to the *CALB2* promoter region included proteins implicated in transcription/translation such as eukaryotic initiation factors 4A-I and –III (*IF4A1*, *EIF4A3*) and elongation factors 1-alpha (*EF1A1*) and 1-delta (*EF1D*) (Fig. 3a, b). Differentially expressed proteins (± Bt) consisted of annexin A1 (*ANXA1*; gene ID: 301; M_r_ 38.7 kDa; − 19%). Even lower amounts of septin 7 (*SEPT7*; gene ID: 989; M_r_ 50.7 kDa; − 34%), a member of the family of GTP-binding proteins and serpin H1 (*SERPH*; − 54%), also known as heat shock protein 47 (HSP47), reported to function as a chaperone for collagen [27], were present in the extracts of Bt-treated cells. Other proteins including annexin A2 (*ANXA2*; gene ID: 302; M_r_ 38.6 kDa) and plasminogen activator inhibitor 2 (*SERPINB2*; gene ID: 5055; M_r_ 47 kDa) were not differentially bound (Fig. 3b). Of interest, the mesenchymal marker vimentin was also present in the complex bound to BRE9–13 DNA and binding was increased in the Bt-treated MSTO-211H samples (+ 44%). Since the decrease in BRE9–13 DNA-bound proteins including annexin A1 and septin 7 could be the result of a decreased binding or of a Bt-induced decrease in protein expression, levels of annexin A1 and septin 7 were determined by Western blot analysis of either cytosolic proteins or proteins eluted from the BRE9–13 DNA fragment in control and Bt-treated MSTO-211H cells. Expression levels of annexin A1 were increased in Bt-treated cells, while the amount of annexin A1 bound to BRE9–13 DNA was nearly unaffected by Bt. On the other hand, cytosolic septin 7 levels were slightly decreased by the Bt treatment in these conditions, while the amount of septin 7 bound to BRE9–13 DNA was clearly lower after Bt treatment (Fig. 3c). To further demonstrate the specificity of the effect, similar experiments were carried out with the previously described DNA fragment containing BRE5–6, shown to act as a Bt-responsive repressor element in colon cancer cells [15]. In parallel, we compared binding of annexin A1 to both BRE-containing fragments, BRE5–6 and BRE9–13 using cytosolic extracts from control or Bt-treated MSTO-211H cells (Fig. 3d). Elution of bound proteins was carried out in 2 conditions: weakly bound proteins eluted in fraction E1 and strongly bound proteins in fraction E2. Since no striking qualitative differences were observed with respect to proteins eluted in fractions E1 and E2, the results from elution conditions E1 and E2 were combined. Similar as shown in Fig. 3c, the signal for septin 7 eluted from immobilized fragment BRE9–13 was clearly weaker using extracts from Bt-treated cells. Yet no significant differences were observed when using DNA fragment BRE5–6 as bait. An inverse situation was observed for annexin A1: increased binding to fragment BRE5–6 using extracts of Bt-treated cells and no striking changes in the binding to fragment BRE9–13 (Fig. 3d). In summary, annexin A1 preferentially bound to the DNA fragment BRE5–6 in a Bt-dependent manner, while septin 7 showed a Bt-sensitive decrease in binding to BRE9–13. Since BRE9–13 acted as a Bt-sensitive activator of *CALB2* promoter activity and septin 7 bound to BRE9–13 was found to be decreased in extracts from Bt-treated cells, we reasoned that septin 7 might be a negative transcriptional regulator of CR expression by its binding within a protein complex that binds to the *CALB2* promoter in MSTO-211H cells.

### Calretinin and septin 7 act as negative transcriptional regulators of each other in MM cells and are inversely regulated by Bt

**Fig. 4:**
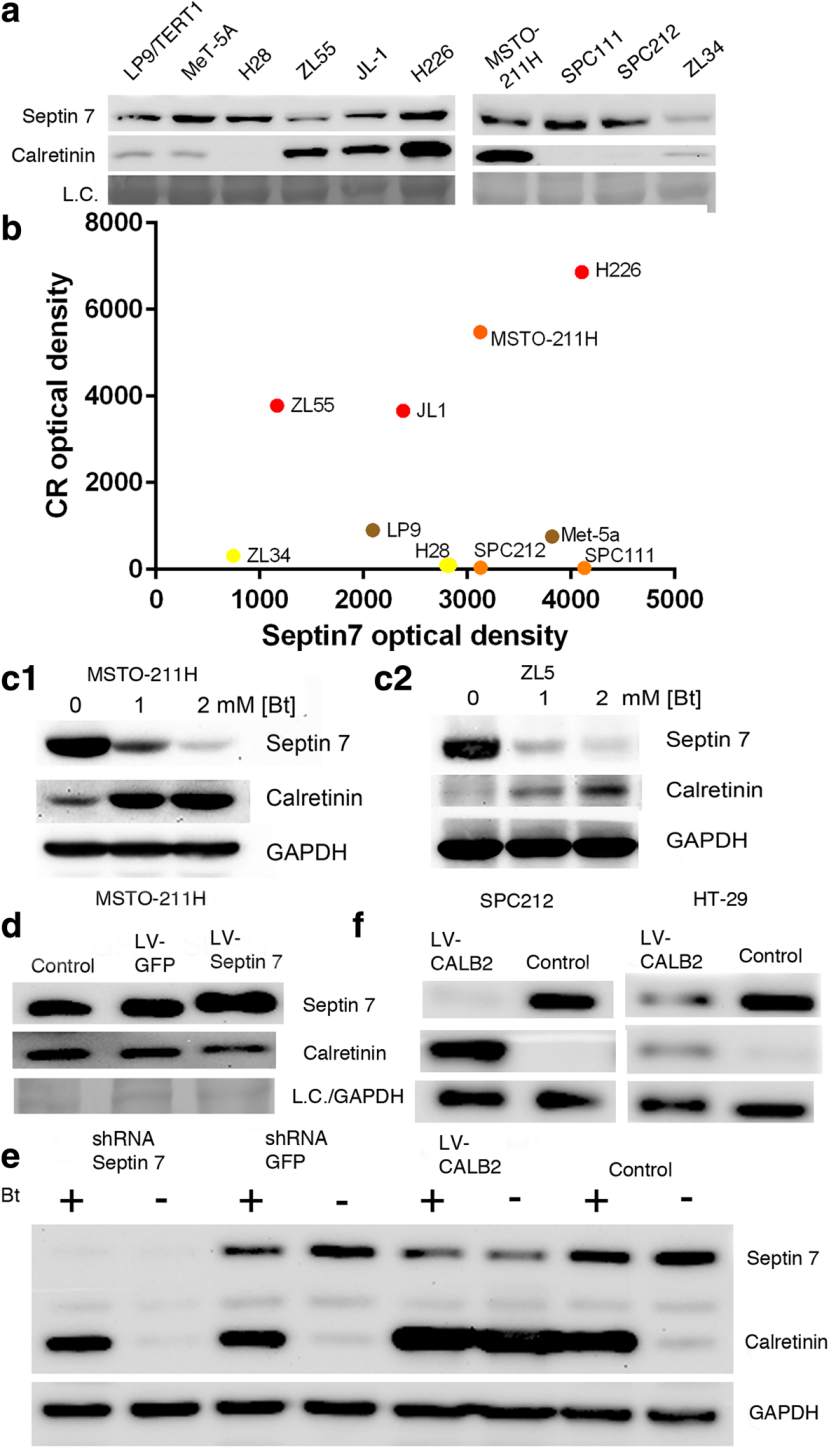
Analysis of septin 7 and CR levels in different cell lines of mesothelial origin and after experimental manipulations. **a** Relative expression levels of CR and septin 7 in various cell lines of mesothelial origin. **b** Semi-quantification of CR and septin 7: brown dots represent immortalized mesothelial cells, red dots epithelioid MM, orange dots bi-phasic and yellow dots sarcomatoid MM cell lines. **c** Effect of Bt administration on CR and septin 7 levels in MSTO-211H (C1) and ZL5 cells (C2). **d** Extracts of MSTO-211H cells infected with LV-septin7 or LV-GFP; note the decrease in CR in cells overexpressing septin 7. **e** MSTO-211H cells treated with Bt (+) or not (−) and subjected to down-regulation of septin 7 by shSEPT7; shGFP served as a negative control. **f** Up-regulation of CR by LV-CALB2 in SPC212 and HT-29 cells; note the decrease in septin 7 levels. The complete Ponceau-stained membranes corresponding to the Western blots shown in **a** and **d** are shown in Additional file 1: Figure S3

First, we investigated CR and septin 7 expression levels in immortalized human mesothelial cells and in human MM cell lines (Fig. 4a). Plotting CR vs. septin 7 levels of all tested cell lines revealed a clear clustering: epithelioid MM (ZL55, ZL5, JL1 and H226) and biphasic MM cells with a major part of epithelioid cells (MSTO-211H) formed the group of high CR-expressing cells (Fig. 4b). Somewhat lower CR levels were found in immortalized mesothelial cells (MeT-5A and LP9/TERT1), followed by the biphasic MM lines with mostly sarcomatoid cells (SPC111, SPC212 and H28) and sarcomatoid cells (ZL34). A clear inverse correlation was also detected in Bt-treated MM cells, where CR and septin 7 levels were determined after 72 h of Bt (1 & 2 mM) treatment. In MeT-5A, ZL55, ZL5, SPC212, ZL34 and MSTO-211H cells CR levels increased (as also shown in Fig. 1), while at the same time septin 7 was clearly down-regulated; representative examples of ZL5 and MSTO-211H cells are shown in Fig. 4c1 and c2. Since these results merely indicated inverse regulation between CR and septin 7 expression by Bt in all investigated MM lines, the next experiments were aimed to alter either CR or septin 7 expression levels to determine the effect on the expression levels of the other protein.

Thus, to directly investigate whether septin 7 acts as a negative transcriptional regulator of CR expression, septin 7 levels were increased by lentiviral infection of MSTO-211H cells with LV-SEPT7. The increase in septin 7 levels led to a down-regulation of CR expression (Fig. 4d), consistent with the previous results that lower amounts of septin 7 bound to BRE9–13 after Bt treatment are correlated with increased CR expression (Fig. 3c). As a negative control, a lentivirus expressing GFP had no effect on CR expression levels. The opposite, i.e. down-regulation of septin 7 by SEPT7 shRNA strongly inhibited cell proliferation, decreased viability and many dying non-adherent cells were observed; the surviving cells had mostly a spindle-shaped morphology typical for the low CR-expressing subpopulation with sarcomatoid morphology of MSTO-211H cells (not shown). While the Bt-induced increase in CR was also seen in shSEPT7-treated cells, septin 7 down-regulation did not noticeably affect CR levels in the surviving MSTO-211H cell population when compared to levels in cells infected with GFP shRNA (Fig. 4e). Since the Bt-induced increase in CR was associated with a decrease in septin 7 as evidenced in control and GFP shRNA treated MSTO-211H cells (Fig. 4e) indicative of a negative feedback regulation, we investigated the direct effects of CR overexpression on septin 7 levels. Lentivirus-mediated upregulation of CR using previously developed tools [25] caused a clear decrease in septin 7, irrespective, whether MSTO-211H cells were treated with Bt or not (Fig. 4e). This indicates that CR is a Bt-independent, negative regulator of septin 7. The inverse experiment with shCALB2 was not conclusive, since shCALB2-mediated down-regulation of CR resulted in massive cell death as reported before [3], thus not allowing to investigating septin 7 levels.

The effect of CR overexpression on septin 7 levels was investigated in SPC212 cells, as well as in the CR-expressing colon carcinoma cell line HT-29, previously shown to negatively modulate CR expression in a Bt-dependent way [15]. In both cell lines, CR overexpression led to a clear decrease in septin 7 levels (Fig. 4f). This supports the hypothesis that CR functions as a negative regulator for septin 7 in two different cell types (mesothelial cells, colonocytes). In summary, treatment with Bt increases CR levels in all histotypes of MM cells and decreases septin 7 levels (data shown for MSTO-211H and ZL5 cells). Finally, overexpression of either one decreases expression levels of the other. These results are indicative of a finely tuned balance between expression levels of the 2 proteins.

### Calretinin and septin 7 are co-expressed during mouse embryonic development and septin 7 levels are higher in primary mesothelial cells from mice without a functional Calb2 gene

**Fig. 5:**
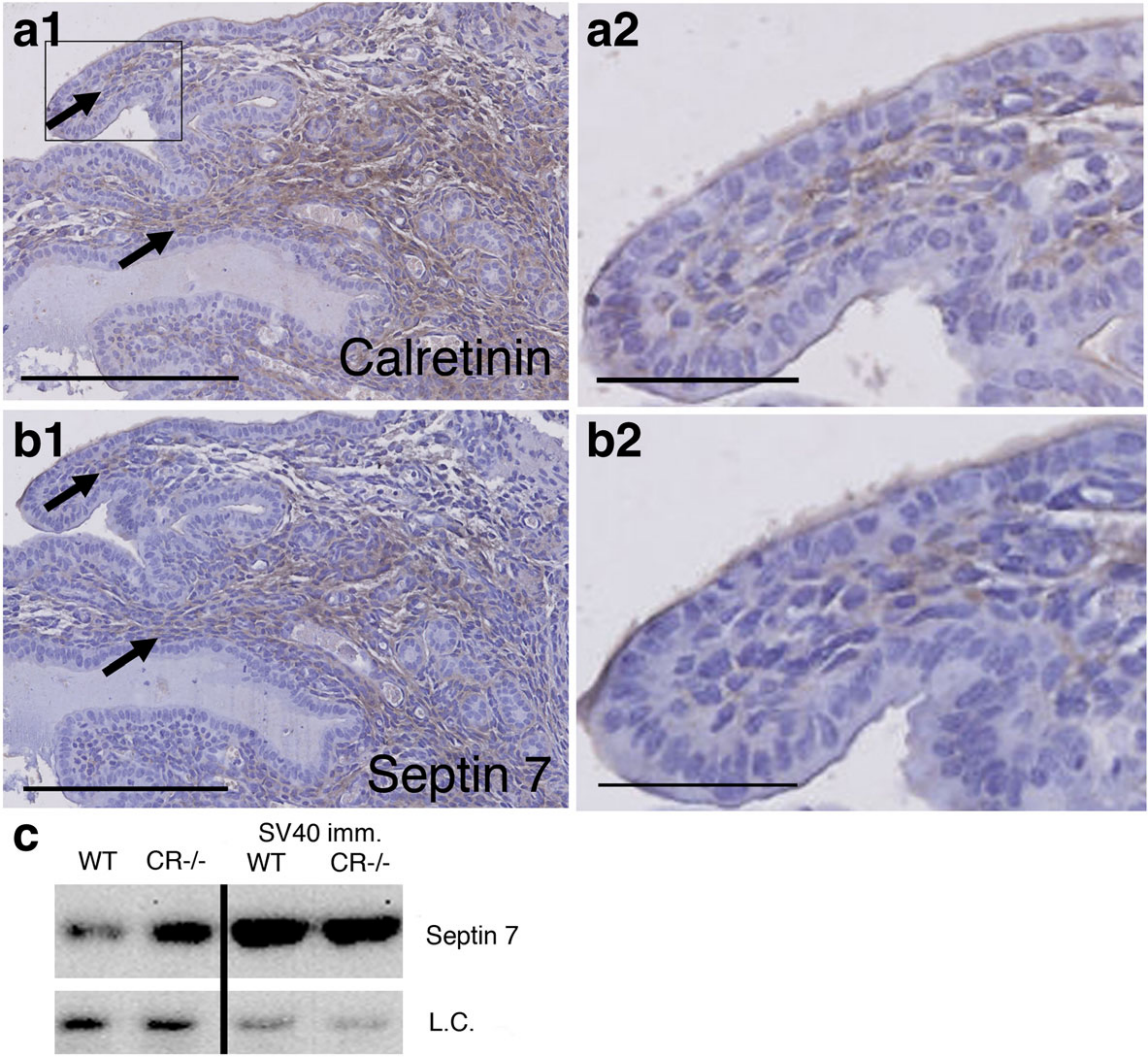
Co-expression of calretinin and septin 7 Immunohistochemical analysis of E10.5 lung tissue stained for CR **a1** and **a2** and septin 7 **b1** and **b2**. Scale bars: 250 μm for **a1** and **b1**, and 50 μm for **a2** and **b2**. **c** Left: Septin 7 Western blot of extracts from cultured primary mesothelial cells from C57Bl/6J (WT) and CR−/− mice; note the lower levels of septin 7 in WT cells. Right: Septin 7 Western blot from SV40-immortalzed mesothelial cells derived from WT and CR−/− mice. As loading control (l.c.) an endogenous biotinylated protein (M_r_ ≈ 75 kDa) was used. The complete Ponceau Red-stained membrane is shown in Additional file 1**:** Figure S3

Based on previous findings that CR is expressed in specific regions within the mesenchyme of murine embryos and in precursor mesothelial cells in the developing lung at embryonic days 14.5 and 16.5 [25], we investigated the expression of CR and septin 7 on serial sections derived from mouse embryos at E10.5. Expression patterns for CR and septin 7 showed an almost complete overlap in the mesenchyme of E10.5 mice (Fig. 5a, b); yet the intensity of staining for one or the other protein varied noticeably. Since transient CR expression is observed in the mesenchyme and developing mesothelial cells [25], we wondered whether CR’s absence during this period in CR−/− mice would affect the expression of septin 7 in the terminally differentiated mesothelium. For this, primary mesothelial cells (prMC) were isolated from either WT or CR−/− mice and kept in cell culture in vitro for 10–15 days. Western blots revealed clearly higher levels of septin 7 in mesothelial cells from CR−/− mice compared to WT animals (Fig. 5c). Thus, the transient expression of CR in developing mesothelial cells had a long-lasting effect on septin 7 expression; it appeared that CR acted as a long-lasting repressor of septin 7 synthesis. A similar analysis was carried out in primary mesothelial cells immortalized with SV40. The expression of SV40 Tag/tag was previously shown to substantially increase the proliferation rate of primary mesothelial cells, both from WT and CR−/− mice [25]. Interestingly, in SV40-immortalized cells, septin 7 expression levels were strongly increased independent of the genotype (WT vs. CR−/−; Fig. 5c**,** right panel).

### Binding of septin 7 to calretinin and co-localization of the two proteins in distinct regions of the cleavage furrow and in the midbody region during cytokinesis

**Fig. 6:**
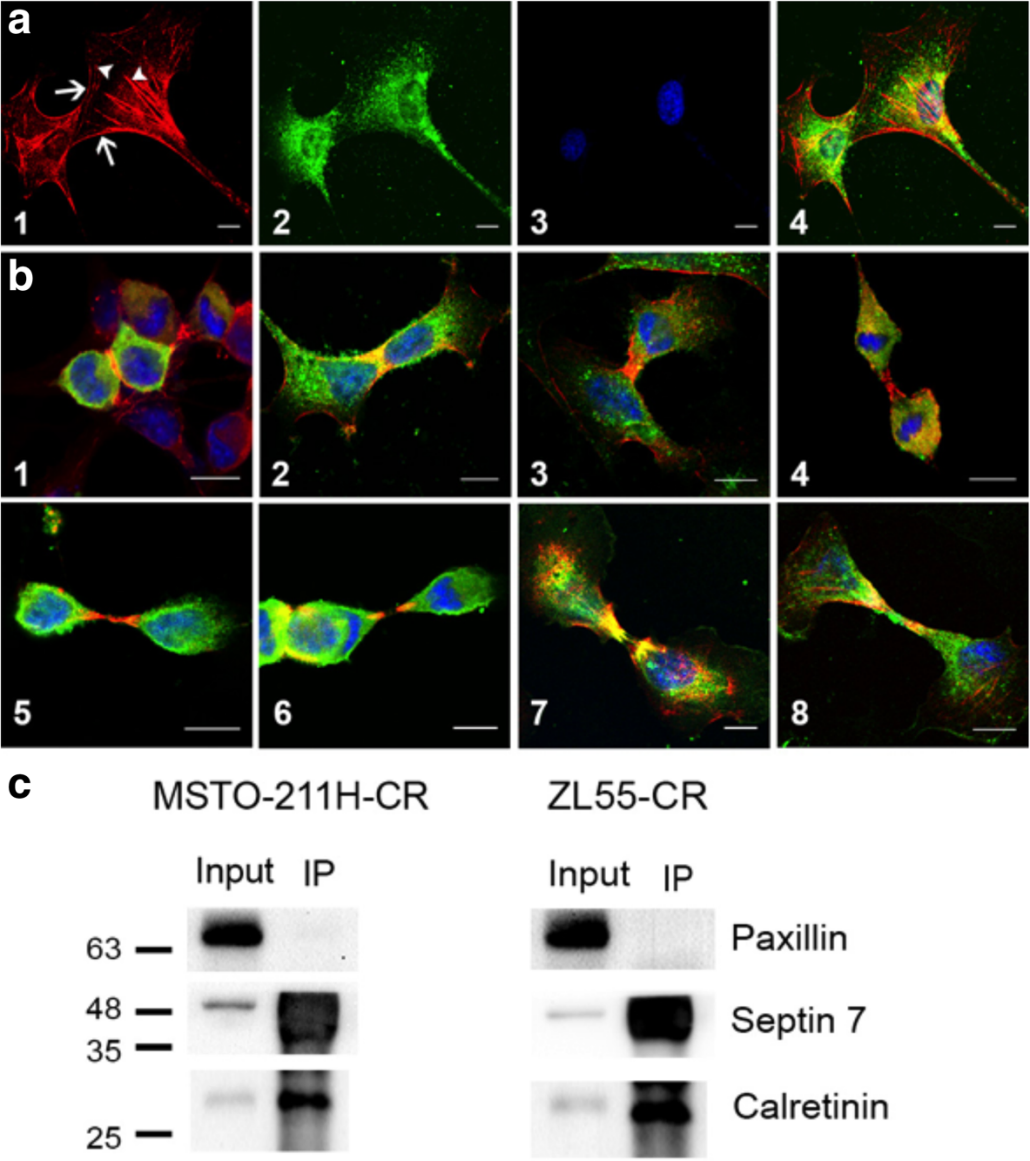
Intracellular localization of calretinin and septin 7 in MM cells **a** Septin 7 localization (red; **a1**) and CR (green; **a2**) in MSTO-211H cells treated with 1 mM Bt. Cortical regions (arrows) and cytoskeletal-like structures (filaments, bundles; arrowheads) are strongly stained for septin 7. **a3** Nuclear DAPI staining. **a4**) Merged images **a1**–**3**). Scale bars: 10 μm. **b** Localization of septin 7 (red) and CR (green) at different stages of cytokinesis starting from early telophase **b1** until close to cell separation **b8**] in MM cell lines. Yellow indicates colocalization of the 2 proteins. DAPI-stained nuclei are shown in blue. Scale bars in all images: 10 μm. 1) MSTO-211H-CR; 2) MSTO-211H treated with 1 mM Bt; 3) MSTO-211H-CR; 4) MSTO-211H treated with 1 mM Bt; 5) MSTO-211H-CR; 6) SPC111-CR; 7) ZL55-CR; 8) SPC212-CR. **c** Co-IP with lysates from MSTO-211H-CR and ZL55-CR cells. Proteins binding to CR were co-immunoprecipitated with a CR antibody. Membranes with PAGE-separated proteins were probed for CR and septin 7 (lane IP). Input samples containing all proteins (prior to immunoprecipitation) were probed for the same proteins. Paxillin was used as negative control

Based on the co-expression of CR and septin 7 in cells from embryonic mesenchymal tissue, we investigated in more detail, the intracellular localization of the two proteins in human MM cells. Since CR immunostaining was rather weak in most untreated MM cell lines, immunofluorescence staining against CR was mostly carried out in cells overexpressing CR or in Bt-treated cells characterized by elevated CR expression levels. Qualitatively, CR intracellular localization was similar in all three conditions: control, CR-overexpressing, Bt-treated cells (data not shown). Septin 7 was either confined to the cell cortex, a zone implicated in the dynamic interplay between plasma membrane proteins and the cytoskeleton and to filamentous structures, likely the so-called “septin cytoskeleton” consisting of septin hetero-polymers (Fig. 6a1). In the same cell (e.g. Bt-treated MSTO-211H), the intracellular distribution of CR was rather homogenous (Fig. 6a2), yet with a stronger staining of perinuclear intermediate filaments and sometimes microtubules as reported before in WiDr colon cancer cells [28]. In WiDr cells a direct association of CR with these cytoskeletal structures has been reported previously, also based on co-immunoprecipitation experiments. The merged images including DAPI staining (Fig. 6a4) showed some colocalization (yellow color) that was further investigated in the different MM cell lines. During telophase septin 7 was strongly localized to the zone of the cleavage furrow (Fig. 6b1). Also CR staining was stronger in the region of cell cleavage sometimes resulting in a yellow zone indicating that the proteins were concentrated in this region (Fig. 6b2). During the early phase of cytokinesis the center of the midbody showed strong “septin 7 only” staining (red) (Fig. 6b3–6), followed first by a yellow zone (septin 7 and CR) and finally by a green one (CR only). In some rare cases the center of the midbody was stained for CR only (Fig. 6b8). Based on the co-localization of these proteins in some regions, we determined whether these two proteins directly interact. Co-immunoprecipitation revealed that in the lysate from MSTO-211H-CR and ZL55-CR cells incubated with anti-CR antibody, septin 7 was pulled down (Fig. 6c). These results suggested that CR and septin 7 have the predisposition to interact directly, but that other putative binding partners of either CR or septin 7 or different conformations of either protein prevented this interaction to take place throughout the cell. Their close spatial apposition (at times even co-localization indicative of direct interaction) and the precise time points during cytokinesis indicates that the 2 proteins are likely implicated in the same biological process, namely in cytokinesis, yet have on occasion distinct localizations and thus probably non-identical, but conceivably complementary functions (see discussion).

## Discussion

Although CR is currently used as a positive marker for the unequivocal identification of human MM of the epithelioid and mixed type [1, 2], still little is known I) about whether CR is implicated in the etiology of MM, II) on the regulation of CR in cells of mesothelial origin and III) about the function(s) of CR in MM cells. Details on the regulation [14] and possible function [3] of CR in MM are only slowly emerging. We had previously reported that Bt acts as repressor of CR expression in CR-positive colon cancer cells via BREs present in the *CALB2* promoter [15]. The same elements BRE5–6 were found to be essentially non-functional in the immortalized mesothelial cell line Met-5A.

We had hypothesized that injury of the intestinal wall or damage of the lung tissue by e.g. asbestos fibers might lead to the colonization of the tunica serosa of the peritoneal or pleural cavity by facultative anaerobic bacteria (e.g. *Streptococcus pneumoniae, Staphylococcus aureus, Chlamydia pneumoniae* in airways or gut microbiota) producing Bt resulting in concentrations in the millimolar range [19]. Thus, we tested the effect of Bt on CR expression levels in immortalized mesothelial cells and several human MM cell lines of all histotypes, the former serving as a model for “reactive” mesothelial cells. The observed strong, rapid and Bt-concentration dependent increase in CR expression depended on the presence of a positive regulatory element in the *CALB2* promoter containing additional BREs. Unexpectedly, septin 7, generally considered as a structural protein was found to be part of this regulatory transcriptional complex. Septin 7 belongs to a family of RAS-like GTP-binding and membrane-interacting proteins; currently 13 septin genes subdivided into four distinct groups are known in mammals and septin family members are characterized by a highly conserved domain structure [29]. The different septins are involved in various cellular processes mostly implicated in cell morphology dynamics including cytoskeleton organization, cytokinesis and membrane dynamics (e.g. curvature), for a review see [29]. Besides septins’ well-characterized preference for protein-protein interactions among own family members, forming higher-order structures such as filaments, bundles, scaffolding structures or rings, many other septin-interacting proteins have been identified forming the septin interactome (see Table 1 in [30]). In many cases these proteins are associated with the actin and/or microtubule cytoskeleton or with phospholipid membranes. Interestingly, the cdk1-dependent phosphorylation of septin 9 regulates the association with the proline isomerase (Pin1) implicated in the disjunction of daughter cells. The observed strong staining of septin 7 at the site of daughter cell separation indicates that septin 7 might have a similar function in MM cell cytokinesis. In support, septin 7-deficient fibroblasts show defects in the machinery implicated in cytokinesis including stabilization of microtubules and stalled midbody abscission [31]. The latter defect was shown to also lead to multinucleation.

Knowledge on the role of septins in gene regulation is sparse. Septin 9 is capable of interacting with the hypoxia-inducible factor 1 alpha HIF-1α acting as a positive regulator in the hypoxic pathway leading to increased proliferation, soft agar clonal survival and tumor growth [32]. In our study we observed that binding of septin 7 to the transcriptional complex driving *CALB2* expression was decreased after Bt treatment. Since the Bt-dependent decrease in septin 7 binding to the protein complex bound to *CALB2* BRE7–13 leading to increased CR levels might be the result of different processes, we selectively manipulated septin 7 expression in MM cell lines. Of note, Bt treatment had no effect on septin 7 expression levels and moreover down-regulation of septin 7 levels by shSEPT7 had no effect on CR expression. On the other hand overexpression of septin 7 led to a strong decrease in CR expression levels indicating that septin 7 acts as a negative regulator (repressor) of CR expression. The mechanism was operating bi-directionally, i.e. CR overexpression resulted in a decrease in septin 7 levels. In line, a comparison of primary mesothelial cells (prMC) from WT and CR−/− mice revealed higher septin 7 levels in CR−/− prMC, albeit the fact that in these cells isolated from young adult mice, CR expression levels are below the detection limit of Western blot analysis [25].

In summary, these results indicate that both, CR and septin 7 act as negative transcriptional regulators for each other. As CR expression levels in tumor specimen are used to diagnose the clinical outcome, i.e. lower CR levels are a poor prognostic factor [33], also deregulated septin expression has been linked to tumor development/growth [34]. Increased levels of septin 2, 8, 9, 11 and decreased levels of septin 4 and 10 have been consistently reported in various tumor types and are assumed to act as oncogenes and tumor suppressor genes, respectively [34]. Evidently in order to exert an antagonistic regulatory function, CR and septin 7 need to be co-expressed in situ, i.e. within the same cells. This was clearly evident during normal mouse embryonic development, where the two proteins colocalize in mesenchymal cells during lung development. While our experiments are in clear support of an inverse regulation, the colocalization and/or close apposition of each other might hint towards an implication in the same process, i.e. in cytokinesis. Immuno-EM images of WiDr colon cancer cells stained with an anti-CR antiserum had previously revealed CR staining during early telophase at the midbody, while at later stages the midbody zone was completely negative (see Fig. 2f-i in [35]). Essentially identical findings on CR localization during cytokinesis were observed in various MM cell lines. Also septin 7 staining was particularly strong first at the cleavage furrow and then in distinct parts of the midbody. Of note, strong co-localization was not observed during the entire separation of the daughter cells; at distinct time points and precise midbody localization one or the other protein was more prevalent resulting in a clear separation of the green and red fluorescence. Yet occasionally, the strong yellow color indicated the 2 proteins to be colocalized at the same regions and co-IP experiments confirmed a direct interaction between CR and septin 7.

Septins have been also shown to associate to the mitotic spindle, being required for cytokinesis [31]; the same had been reported for CR-expressing WiDr cells [35, 36].

## Conclusions

In this report we have identified septin 7, an essential cellular component implicated in the final steps of cell division, as a strong Bt-dependent gene regulatory protein binding to the promoter of *CALB2*. Moreover, septin 7 was negatively regulated by CR forming a feedback loop. Our study adds knowledge on the molecular mechanism involved in up-regulation of CR caused by Bt, a step that might also be implicated in mesotheliomagenesis. Since CR has been proposed to serve as a putative target for MM therapy due to its essential role in MM cell lines [3], indirectly targeting CR through septin 7 and/or directly targeting septin 7 might represent yet another strategy for the development of a therapy to treat the currently incurable MM.

## Additional file

Additional file 1: **Figure S1.** MTT assay of different MM cell lines exposed to butyrate (Bt). Relative MTT signals for A) MSTO-211H, B) ZL55 and C) ZL5 MM cells exposed to various Bt concentrations ranging from 0.33 to 5 mM. Results are from 3 independent experiments (each sample in triplicate). The value of untreated cells in each experiment was defined as 100%. Results represent mean±SEM. **Figure S2.** Point mutations in the PubMed database sequence (*CALB2*; gene ID: 794) of the *CALB2* promoter region containing BRE7-13 in comparison to human Met-5A and ZL55 cells are boxed in green (Met-5A) or yellow (ZL55). Insertions or deletions found in the sequence of all analyzed cell lines are boxed in cyan. None of the mutations concern the 7 BRE listed in Fig. 2. **Figure S3.** *Top panel*: Ponceau-Red stained membrane used for the Western blot shown in Fig. 4a. Sizes of marker proteins range from 17 kDa (faintly stained lowest band marking gel front) to 100 kDa (most upper band). Middle panel: Ponceau-Red stained membrane used for the Western blot shown in Fig. 4d. The size of marker proteins ranges from 20 kDa to 135 kDa. Lower panel: Ponceau-Red stained membrane used for the Western blot shown in Fig. 5c. The size of marker proteins ranges from 20 kDa to 135 kDa. (DOCX 1083 kb)

## Abbreviations

BRE: Butyrate-responsive elements
Bt: Butyrate
CMF: Calcium- and magnesium-free
CR: Calretinin
E10.5: Embryonic day 10.5
HRE: Hypoxia-responsive elements HRE
MM: Malignant mesothelioma
prMC: Primary mesothelial cells
