# Supplementary material for: Regulation of calretinin in malignant mesothelioma is mediated by septin 7 binding to the CALB2 promoter
Source: BMC Cancer. 2018 Apr 27;18:475. doi: 10.1186/s12885-018-4385-7 (PMC5922012; doi:10.1186/s12885-018-4385-7)
Supplement: Supplementary file 1 — Figure S1. MTT assay of different MM cell lines exposed to butyrate (Bt). Relative MTT signals for A) MSTO-211H, B) ZL55 and C) ZL5 MM cells exposed to various Bt concentrations ranging from 0.33 to 5 mM. Results are from 3 independent experiments (each sample in triplicate). The value of untreated cells in each experiment was defined as 100%. Results represent mean±SEM. Figure S2. Point mutations in the PubMed database sequence (CALB2; gene ID: 794) of the CALB2 promoter region containing BRE7-13 in comparison to human Met-5A and ZL55 cells are boxed in green (Met-5A) or yellow (ZL55). Insertions or deletions found in the sequence of all analyzed cell lines are boxed in cyan. None of the mutations concern the 7 BRE listed in Fig. 2. Figure S3. Top panel: Ponceau-Red stained membrane used for the Western blot shown in Fig. 4a. Sizes of marker proteins range from 17 kDa (faintly stained lowest band marking gel front) to 100 kDa (most upper band). Middle panel: Ponceau-Red stained membrane used for the Western blot shown in Fig. 4d. The size of marker proteins ranges from 20 kDa to 135 kDa. Lower panel: Ponceau-Red stained membrane used for the Western blot shown in Fig. 5c. The size of marker proteins ranges from 20 kDa to 135 kDa. (DOCX 1083 kb) [file 12885_2018_4385_MOESM1_ESM.docx]

**Additional files**

**
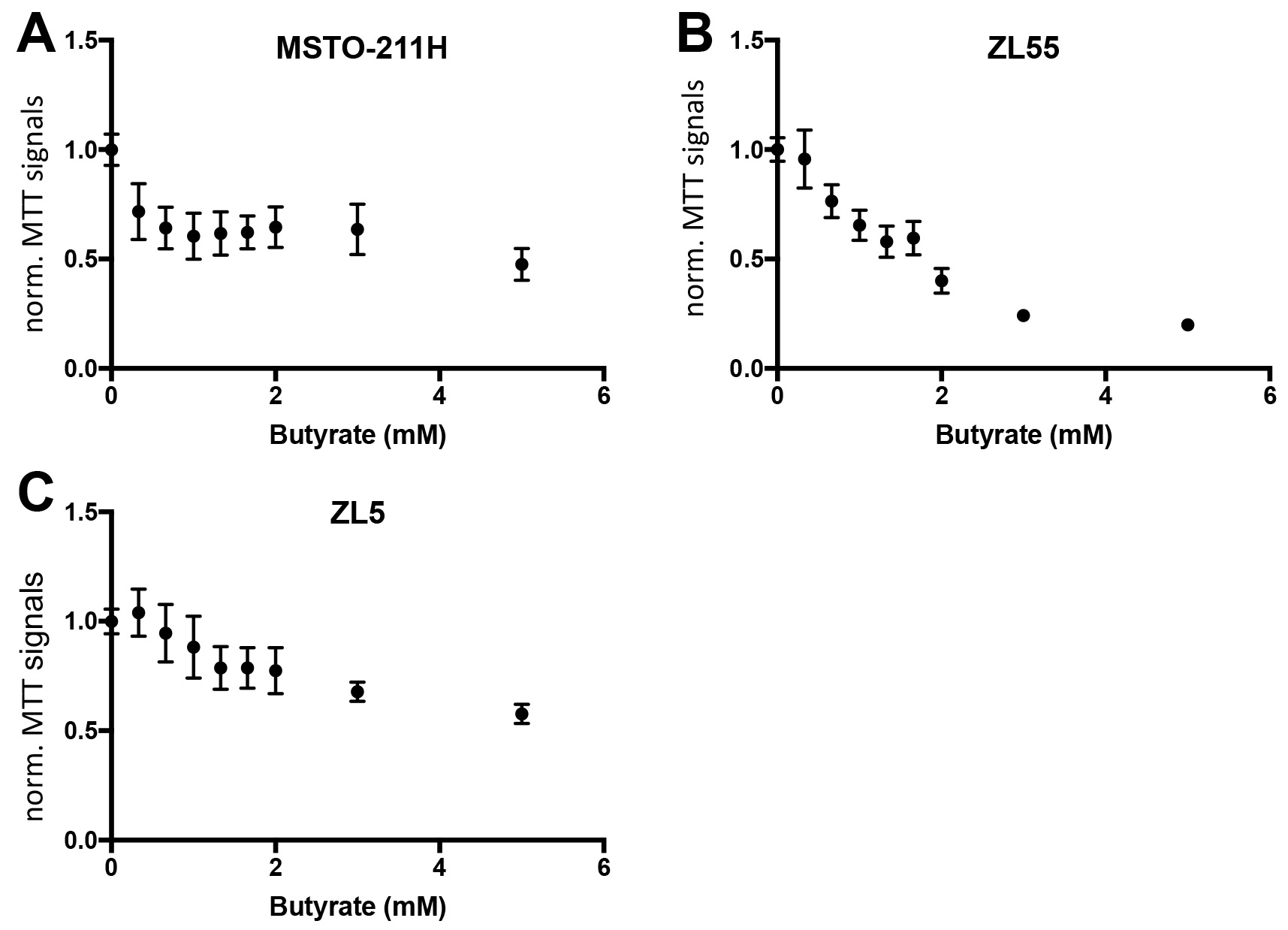
**

**Fig. S1 MTT assay of different MM cell lines exposed to butyrate (Bt).** Relative MTT signals for **A)** MSTO-211H, **B)** ZL55 and **C)** ZL5 MM cells exposed to various Bt concentrations ranging from 0.33 to 5 mM. Results are from 3 independent experiments (each sample in triplicate). The value of untreated cells in each experiment was defined as 100%. Results represent mean±SEM.

**Fig. S2 Sequence comparison of *CALB2* promoter regions containing BRE7-13**

**CALB2 sequence from PubMed**

atgcgaaagtgaaggtggtccccagtttgcccttctggtggagggaggaaccagccatcagagaactggtgggtaaacagtgggcttggcattactccttaacgctgggaggggccctgtggctccagtggccagggtccgggaagaactttatacatagtgcccaggagcttggcacctggtccacaaagactccatataccatggaaactggaggggacaaagtgttcttggtaccccacccctcccaggtcttggttgggtgttaatctctctgagccttgatttccctatctttggaatgggtcaaattcttgcattgcacacctgataggggagttgtgaggaccaaacaaagcaagacacatcaaaaccctctgtaaaggcatttaaaggtatcctcacggttatcgccatcattgtcgtcatcatcatcattatcattattgtaataaaactcgaggggaagtcctggaagggaagcagaaccccagacatctgaaattacttacaagagccttaattcacggaaatgtgctcataaatgttccgtctttaccgtcactgctttaaaatacgctcatcgaacctctctttcctatctctgaatgatttcttatctgagtccatgtttctcctcacacatttggttattgaaacttggaccagtccagctaagactccag_ctctcctccaatcgaaacgtcttcctccctttcccattgcaaactggaacaaggagcttgggctggagctgagcctggagagaagggatggctggctctaagacatcccctcctggtagggcagagggagctgcaaggagagatgaggggcgggaggtcttcctacctctctggtcgcttgcagggttgtaagagcatcccaggctttggctcccgctgctgatctctgaggaatttgagggcctgtgcatagtggagtgaaggttttttggagcggtcttcagctccgctctagcttcctcaacaccgaggatatcataaacaccagcaggtatctggcttcggccccctctttaaactatatgcacctttcccaggagccatagcaacccccaagggctgccgagaaccatggtggctcccatcttccttctagacgcaggacagggcagtctaagctgctttcataggccatcagacagctctgagtcagaagcaagcgccagtcccaactccatatgtggcccaaactccagaacactgctcaccccagcccactctggagccaggtgacatctccagttcctccttgct

**CALB2 sequence from Met-5A cells**

ATGCGAAAGTGAAGGTGGTCCCCAGTTTGCCCTTCTGGTGGAGGGAGGAACCAGCCATCAGAGAACTGGTGGGTAAACAGTGGGCTTGGCATTACTCCTTAACGCTGGGAGGGGCCCTGTGGCTCCAGTGGCCAGGGTCCGGGAAGAACTTTATACATAGTGCCCAGGAGCTTGGCACCTGGTCCACAAAGACTCCATATACCATGGAAACTGGTGGGGACAAAGTGTTCTTGGTACCCCACCCCTCCCAGGTCTTGGTTGGGTGTTAATCTCTCTGAGCCTTGATTTCCCTATCTTTGGAATGGGTCAAATTCTTGCATTGCACACCTGATAGGGGAGTTGTGAGGACCAGACAAAGCAAGACACATCAAAACCCTCTGTAAAGGCATTTAAAGGTATCCTCACGGTTATCGCCATCATTGTCGTCATCATCATCATTATCATTATTGTAATAAAACTCGAGGGGAAGTCCTGGAAGGGAAGCAGAACCCCAGACATCTGAAATTACTTACAAGAGCCTTAATTCACGGAAATGTGCTCATAAATGTTCCGTCTTTACCGTCACTGCTTTAAAATACGCTCATCGAACCTCTCTTTCCTATCTCTGAATGATTTCTTATCTGAGTCCATGTTTCTCCTCACACATTTGGTTATTGAAACTTGGACCAGTCCAGCTAAGACTCCAGTCTCTTCTCCAATGCAAAGCTCTTC__TCCTTTCCCATTGCAAACTGGA_CAAGGAGCTTGGGTCTGGAGCTGAGCCTGGAGAGAAGGGATGGCTGGCTCTAAGACATCCCCTCCTGGTAGGGCAGAGGGAGCTGCAAGGAGAGATGAGGGGCGGGAGGTCTTCCTACCTCTCTGGTCGCTTGCAGGTTGTAAGAGCATCCCAGGCTTTGGCTCCCGCTGCTGATCTCTGAGGAATTTGAGGGCCTGTGCATAGTGGAGTGAAGGTTTTTTGGAGCGGTCTTCAGCTCCGCTCTAGCTTCCTCAACACCGAGGATATCATAAACACCAGCAGGTATCTGGCTTCGGCCCCCTCTTTAAACTATATGCACCTTTCCCAGGAGCCATAGCAACCCCCAAGGGCTGCCGAGAACCATGGTGGCTCCCATCTTCCTTCTAGACGCAGGACAGGGCAGTCTAAGCTGCTTTCATAGGCCATCAGACAGCTCTGAGTCAGAAGCAAGCGCCAGTCCCGACTCCATATGTGGCCCAAACTCCAGAACACTGCTCACCCCAGCCCACTCTGGAGCCAGGTGACATCTCCAGTTCCTCCTTGCT

**CALB2 sequence from ZL55 cells**

ATGCGAAAGTGAAGGTGGTCCCCAGTTTGCCCTTCTGGTGGAGGGAGGAACCAGCCATCAGAGAACTGGTGGGTAAACAGTGGGCTTGGCATTACTCCTTAACGCTGGGAGGGGCCCTGTGGCTCCAGTGGCCAGGGTCCGGGAAGAACTTTATACATAGTGCCCAGGAGCTTGGCACCTGGTCCACAAAGACTCCATATACCATGGAAACTGGAGGGGACAAAGTGTTCTTGGTACCCCACCCCTCCCAGGTCTTGGTTGGGTGTTAATCTCTCTGAGCCTTGATTTCCCTATCTTTGGAATGGGTCAAATTCTTGCATTGCACACCTGATAGGGGAGTTGTGAGGACCAAACAAAGCAAGACACATCAAAACCCTCTGTAAAGGCATTTAAAGGTATCCTCACGGTTATCGCCATCATTGTCGTCATCATCATCATTATCATTATTGTAATAAAACTCGAGGGGAAGTCCTGGAAGGGAAGCAGAACCCCAGACATCTGAAATTACTTACAAGAGCCTTAATTCACGGAAATGTGCTCATAAATGTTCCGTCTTTACCGTCACTGCTTTAAAATACGCTCATCGAACCTCTCTTTCCTATCTCTGAATGATTTCTTATCTGAGTCCATGTTTCTCCTCACACATTTGGTTATTGAAACTTGGACCAGTCCAGCTAAGACTCCAGTCTCTTCTCCAATGCAAAGCTCTTC__TCCTTTCCCATTGCAAACTGGA_CAAGGAGCTTGGGTCTGGAGCTGAGCCTGGAGAGAAGGGATGGCTGGCTCTAAGACATCCCCTCCTGGTAGGGCAGAGGGAGCTGCAAGGAGAGATGAGGGGCGGGAGGTCTTCCTACCTCTCTGGTCGCTTGCAGGTTGTAAGAGCATCCCAGGCTTTGGCTCCCGCTGCTGGTCTCTGAGGAATTTGAGGGCCTGTGCGTAGTGGAGTGAAGGTTTTTTGGAGCGGTCTTCAGCTCCGCTCTAGCTTCCTCAACACCGAGGATATCATAAACACCAGCAGGTATCTGGCTTCGGCCCCCTCTTTAAACTATATGCACCTTTCCCAGGAGCCATAGCAACCCCCAAGGGCTGCCGAGAACCATGGTGGCTCCCATCTTCCTTCTAGACGCAGGACAGGGCAGTCTAAGCTGCTTTCATAGGCCATCAGACAGCTCTGAGTCAGAAGCAAGCGCCAGTCCCAACTCCATATGTGGCCCAAACTCCAGAACACTGCTCACCCCAGCCCACTCTGGAGCCAGGTGACATCTCCAGTTCCTCCTTGCT

**Fig. S2** Point mutations in the PubMed database sequence (*CALB2*; gene ID: 794) of the *CALB2* promoter region containing BRE7-13 in comparison to human Met-5A and ZL55 cells are boxed in green (Met-5A) or yellow (ZL55). Insertions or deletions found in the sequence of all analyzed cell lines are boxed in cyan. None of the mutations concern the 7 BRE listed in Fig. 2.

**Fig. S3**

#
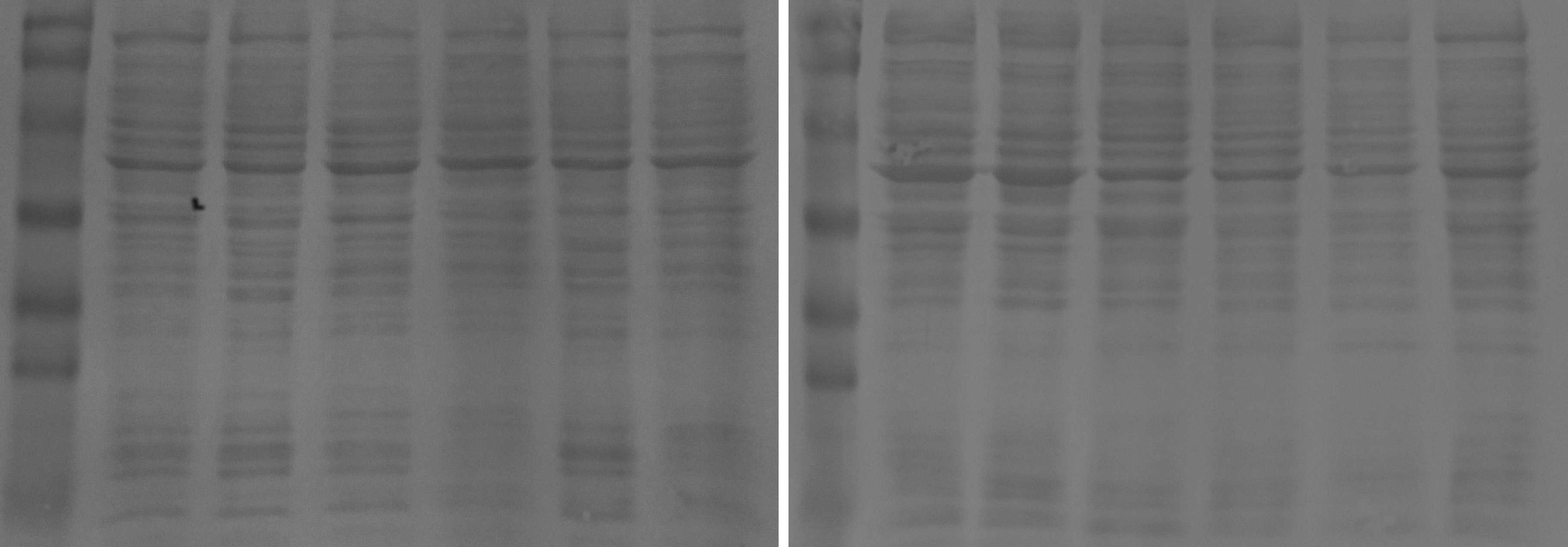


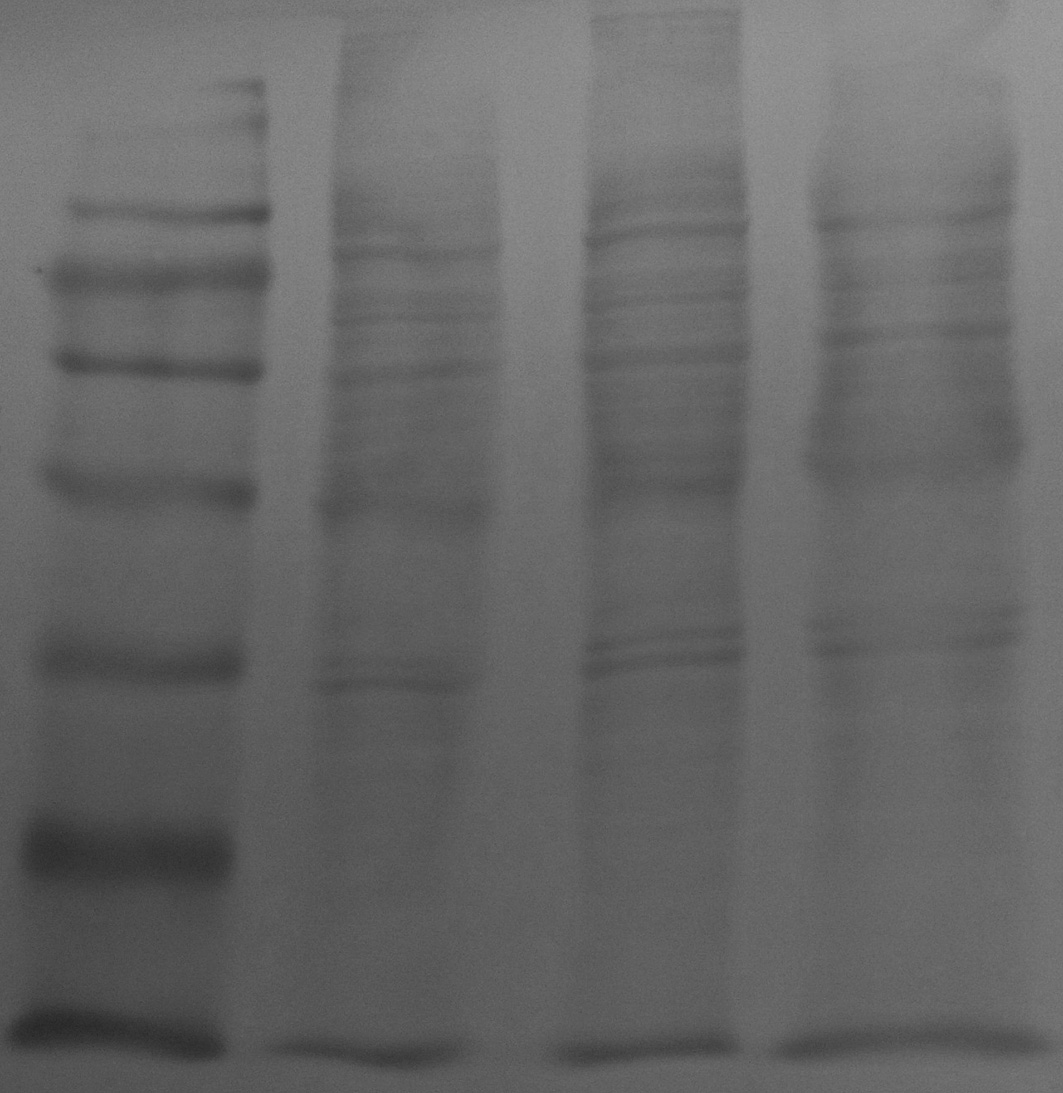


**
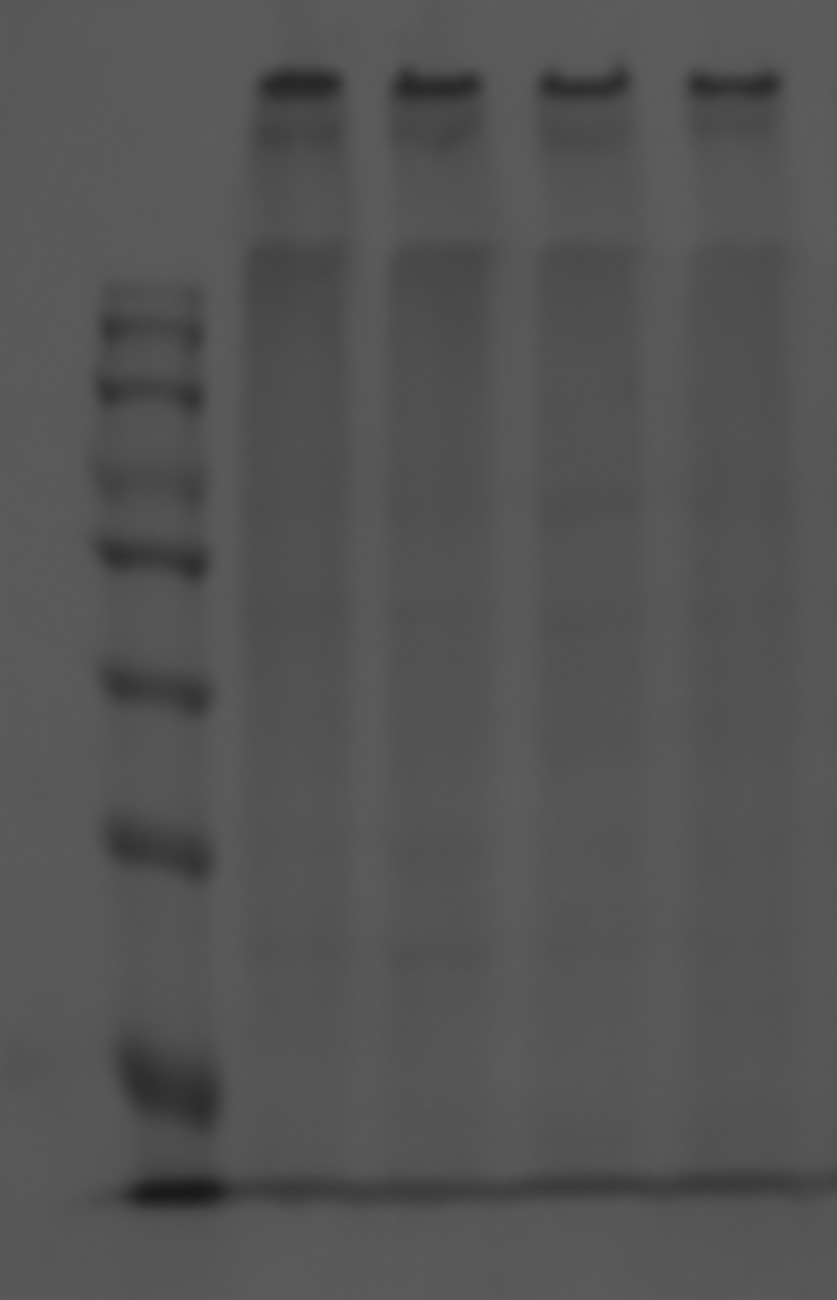
**

**Fig.S3** *Top panel*: Ponceau-Red stained membrane used for the Western blot shown in **Fig. 4A**. Sizes of marker proteins range from 17 kDa (faintly stained lowest band marking gel front) to 100 kDa (most upper band).

*Middle panel*: Ponceau-Red stained membrane used for the Western blot shown **in Fig. 4D**. The size of marker proteins ranges from 20 kDa to 135 kDa. *Lower panel*: Ponceau-Red stained membrane used for the Western blot shown **in Fig. 5C**. The size of marker proteins ranges from 20 kDa to 135 kDa.
